# Identification of Potential Vaccine Strains of *Escherichia coli* Isolates From Diarrhoeic Calves

**DOI:** 10.1002/vms3.71151

**Published:** 2026-08-09

**Authors:** Yasemin Erdoğan, Ediz Kağan Özgen, Murat Özmen, Elif Karadeniz Pütür, Nebahat Polat, Ersan Pütür, Mücahit Harmancı, Ercan Atalay, Ahmet Temur, Meryem Güloğlu, Lütfullah Celep, Seyda Cengiz, Mehmet Cemal Adıgüzel, Cihan Öz, Timur Gülhan, Alper Çiftci, Erhan Çiftgül

**Affiliations:** ^1^ Department of Bacteriology, Erzurum Veterinary Control Institute Republic of Türkiye Ministry of Agriculture and Forestry Erzurum Türkiye; ^2^ Department of Veterinary Microbiology, Faculty of Veterinary Medicine Atatürk University Erzurum Türkiye; ^3^ Department of Microbiology, Milas Faculty of Veterinary Medicine Mugla Sitki Kocman University Muğla Türkiye; ^4^ Department of Veterinary Microbiology, Faculty of Veterinary Medicine Ondokuz Mayıs University Samsun Türkiye; ^5^ Erzurum Directorate of Provincial Agriculture and Forestry Republic of Türkiye Ministry of Agriculture and Forestry Erzurum Türkiye

**Keywords:** Calf diarrhoea, *E. coli*, flow cytometry, immunization, MLVA, antimicrobial resistance

## Abstract

**Background:**

*Escherichia coli* is the most commonly isolated bacterium in cases of diarrhoea in calves under the age of 2 months.

**Objectives:**

This study aimed to identify *E. coli* strains with immunogenic potential by characterizing isolates from diarrheic calves through antigenic profiling and genotyping.

**Methods:**

The phenotypic and genotypic antibiotic resistance characteristics and virulence genes of the *E. coli* strains isolated from 500 faecal samples were examined, of which 88 were further genotyped using the Multiple‐Locus Variable‐Number Tandem Repeat Analysis (MLVA) method. Subsequently, 20 *E. coli* strains were selected based on their antibiotic resistance patterns and genetic typing (MLVA) results, and subjected to an analysis of their outer membrane vesicle profile.

**Results:**

Among these, strains Ec048 and Ec026 exhibited high OMV diversity and were identified as promising candidates for further immunogenic evaluation. Immunization of experimental animals with these strains enhanced humoural immune responses (IgG, IgM, CD20), whereas cellular immune parameters (CD4, CD8) showed no significant change.

**Conclusions:**

In conclusion, the study demonstrates that strains Ec048 and Ec026 elicit strong humoural immune responses, indicating their potential for vaccine development. However, protective efficacy was not assessed, and further studies including challenge experiments are required to confirm their suitability as vaccine strains.

## Graphical abstract

This study aimed to identify vaccine candidate *Escherichia coli* strains isolated from diarrheic calves. Faecal samples collected from 500 calves were analysed, and *E. coli* strains were isolated and characterized phenotypically and genotypically. Antibiotic resistance profiles, virulence‐associated genes and MLVA genotyping were used to select 20 representative strains. These strains were subsequently evaluated for their outer membrane vesicle (OMV) profiles, from which strains Ec048 and Ec026 demonstrated the highest OMV diversity and were identified as promising vaccine candidates.

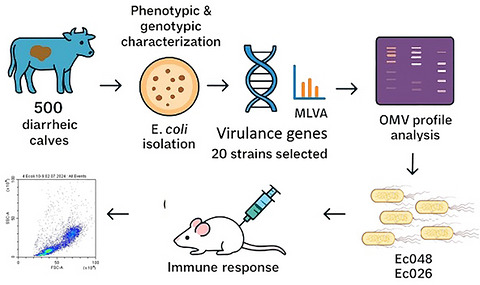

## 1 Introduction

Diarrhoea is a common health problem in calves, starting in the early stages of life and adversely affects the living conditions of animals in nearly all livestock facilities around the world (Al Mawly et al. 2015). Calf diarrhoea continues to be a major economic burden for livestock owners (Kolenda et al. 2015). *Escherichia coli* is the most commonly isolated microorganism in cases of diarrhoea in calves (Mohammed et al. 2019). *E. coli* can be further classified into enterotoxigenic *E. coli* (ETEC), enteropathogenic *E. coli* (EPEC), enterohaemorrhagic *E. coli* (EHEC), Shiga toxin‐producing *E. coli* (STEC), diffusely adherent *E. coli* (DAEC), enteroaggregative *E. coli* (EAggEC), enteroinvasive *E. coli* (EIEC) and necrotoxigenic *E. coli* (NTEC) pathotypes (Özgen et al. 2023).

There are several key virulence factors contributing to the pathogenesis of *E. coli*, including adhesins (*f17* fimbria, S, P fimbria, afimbrial adhesins, capsule‐like adhesin structures), toxins (Shiga toxin, *CNF1, CNF2*, Labile toxin, Stable toxin), aerobactin and outer membrane proteins such as *TraT* and *OmpA* (Kaipainen et al. 2002; Kaper et al. 2004). Advances in molecular techniques have facilitated the identification of the main virulence factors in *E. coli* strains (Güler et al. 2008).

The rapid typing of pathogens and the elucidation of their relationships are essential for effective infection control. Among the methods used for this purpose, Multiple‐Locus Variable‐Number Tandem Repeat Analysis (MLVA) offers highly reliable intraspecies subtyping through an analysis of repeated sequences within the target region (Helldal et al. 2017). MLVA has been reported to facilitate the greater genetic discrimination of *E. coli* strains when compared to Pulsed Field Gel Electrophoresis (PFGE), which is considered the optimum approach in molecular epidemiological studies (Christiansson et al. 2011).

Outer membrane protein structures play a key role in eliciting the immune response against ETEC strains (Roy et al. 2010). The outer membrane proteins of *E. coli*, including outer membrane vesicles (OMVs), enhance the interaction between the bacterium and host cells, and studies have found reductions in these structures to be associated with diminished immune response (Edwards et al. 2011). Earlier studies have also reported an upregulation in the expression of TLR‐related genes of host cells exposed to strains carrying OMV structures as well as changes in the levels of inflammatory and anti‐inflammatory cytokines (Behrouzi et al. 2018). Furthermore, such structures have been identified as promising candidates for use in vaccine studies (Huang et al. 2016).

Given the concerning increase in antibiotic resistance, other disease prevention strategies have been investigated in the fight against infections that could help reduce the prevalence of antibiotic‐resistant strains (Rojas‐Lopez et al. 2018). Vaccination strategies targeting cows or their calves during pregnancy are employed to protect against calf diarrhoea. The antibody content in the maternal colostrum is directly influenced by vaccination during gestation (Maier et al. 2022). Numerous vaccines are available in North America and Europe targeting the mortality and morbidity associated with calf diarrhoea (Kohara et al. 1997). Several studies of calf diarrhoea have reported that vaccinations applied during field trials lack efficacy, underlining the discrepancy between outbreak strains and the dominant pathogenic strains circulating in the field as the leading cause of failure (Maier et al. 2022). There have also been studies recommending the development of vaccines and hyperimmune sera against country‐specific strains, given the broad variety of *E. coli* strains identified in different geographic regions (Coşkun and Şahin 2023).

This study aimed to isolate *E. coli* from faecal samples of diarrheic calves in Eastern Anatolia, Türkiye and to characterize their virulence traits, antibiotic resistance, OMV profiles and genetic relationships in order to identify strains with immunogenic potential for further evaluation in experimental immunization studies.

## 2 Materials and Method

### 2.1 Study Area Description

The study was conducted in an area of approximately 164,000 km^2^, encompassing the provinces of Erzurum, Erzincan, Kars, Ağrı, Ardahan, Bayburt, Artvin, Gümüşhane and Iğdır, where cattle farming is intensive. This area represents the northern and eastern parts of the Eastern Anatolia Region (39°30′ N–43°30′ N, 38°30′ E–44°30′ E), one of Türkiye's most important livestock centres.

The cattle population in the research area consists of over 2.5 million animals distributed across approximately 180,000 farms. The dominant production model in the region is small and medium‐sized farms with an average herd size of 10–25 head. In general, traditional livestock farming is practiced in the region, with a pasture‐based feeding system adopted.

In the study, farm selection was determined randomly based on the presence of diarrheal calves and the voluntary participation of the farmers. To ensure a representative sample across the region, the sample size collected from each province was adjusted proportionally to the cattle population density of that province.

### 2.2 Sampling and Bacterial Culturing

Faecal samples were obtained from 500 diarrheic calves, ranging in age from 1 day to 2 months, by stimulating defecation via digital rectal stimulation. The samples were collected directly into sterile tubes, placed in sterile transfer containers and transported to the laboratory within 4–6 h in a cooling box maintained at 4°C.

The faecal samples collected from the calves were initially inoculated into nutrient broth and then onto MacConkey and tryptone bile x‐glucuronide (TBX) media, before being incubated at 37°C for 24–48 h (Clifton‐Hadley 2000; Markey et al. 2013). Following incubation, *E coli*‐suspected colonies were subcultured onto sheep blood agar to isolate single pure colonies. After single colonies were observed, Gram staining and IMViC tests were performed (Markey et al. 2013). At the end of the analysis, strains identified as *E. coli* were transferred to tryptic soy broth for further incubation, after which, 250 µL aliquots of the liquid culture were transferred into two separate sterile Eppendorf tubes for storage at −80°C. The ATCC 25922 strain was adopted as the positive control for the study.

### 2.3 Antimicrobial Susceptibility Test

The phenotypic antimicrobial susceptibility of the isolated *E. coli* strains was assessed by the disk diffusion method, using agents selected to represent the principal antimicrobial classes, acting through distinct mechanisms of action, that are routinely used to treat neonatal calf diarrhoea in the study region, thereby enabling field‐relevant resistance to be assessed and correlated with the resistance genes screened in this study. In the present study, the isolates were tested for susceptibility and resistance to the following antimicrobial agents: enrofloxacin (5 µg), marbofloxacin (5 µg), amoxicillin/clavulanic acid (30 µg), ampicillin/sulbactam (20 µg), oxytetracycline (30 µg), trimethoprim/sulfamethoxazole (25 µg), ceftiofur (30 µg) and neomycin (30 µg). To this end, the isolates were adjusted to a 0.5 MacFarland standard and 0.1 mL was inoculated onto Mueller–Hinton agar. After inoculation, antimicrobial‐impregnated discs were placed on the agar medium, and all plates were then incubated at 37°C for 24 h (El‐Tayeb et al. 2017). At the end of the incubation period, the susceptibility and resistance of the isolates were evaluated according to the European Committee on Antimicrobial Susceptibility Testing (EUCAST) guidelines.

### 2.4 DNA Extraction

Following the bacteriological culture analyses, 200 µL from each isolate was subjected to DNA extraction using the automated IndiMag system (Indical) using an IndiMag Pathogen IM48 kit, and the extracted total nucleic acids were stored at −20°C prior to their PCR and MLVA analyses. The concentration and purity of the extracted nucleic acids were assessed spectrophotometrically using a NanoDrop microvolume spectrophotometer (Thermo Fisher Scientific, USA); the A260/A280 ratio was used to verify DNA purity (≈1.8–2.0 accepted as pure) and the DNA concentration (ng/µL) was recorded for each sample prior to PCR.

### 2.5 Determination of *E. coli* Virulence Genes

After DNA extraction from *E. coli* isolates, PCR was used to detect the virulence genes stx1, stx2, Intimin, F41, F5 (K99), sta, fimH, traT, ompT, f17A, cnf1, cnf2, cs31A and phoA (Güler et al. 2008; Bertin et al. 1996; Chapman et al. 2006; Johnson et al. 2010; Shome et al. 2011). Primer sequences specific to the virulence genes and the expected PCR product sizes are included in the Supporting Information.

All PCR reactions were performed using the HotStarTaq Master Mix Kit (Qiagen, Germany) according to the manufacturer's instructions. Each reaction was prepared to a final volume of 25 µL, comprising 12.5 µL of 2× HotStarTaq Master Mix (providing HotStarTaq DNA polymerase, PCR buffer, 1.5 mM MgCl_2_ and 200 µM of each dNTP at final concentration), each primer at a final concentration of 0.5 µM, and approximately 100 ng of template DNA, with RNase‐free water added to bring the reaction to its final volume. The prepared reaction tubes were subjected to thermal cycling as specified in Table 1.

**TABLE 1:** Thermal cycling conditions used for the amplification of the virulence factor genes, antimicrobial resistance genes and MLVA loci, according to the respective reference protocols.

Category	Target gene(s)/loci	Initial denaturation	Cycling (No. × [denaturation / annealing / extension])	Final extension	Ref.
Virulence factors	stx1, stx2, intimin (eae), F5 (K99), F41, sta	95°C, 15 min	25 × (94°C 30 s / 50°C 45 s / 72°C 1 min)	72°C, 10 min	Güler et al. (2008)
	f17A	95°C, 15 min	25 × (94°C 1 min / 55°C 1 min / 72°C 1 min)	72°C, 10 min	Bertin et al. (1996)
	fimH, traT, ompT	95°C, 15 min	25 × (94°C 30 s / 63°C 30 s / 72°C 1 min)	72°C, 10 min	Chapman et al. (2006)
	cnf1, cnf2, cs31A	95°C, 15 min	25 × (94°C 30 s / 60°C 30 s / 72°C 1 min)	72°C, 10 min	Johnson et al. (2010)
	phoA	95°C, 15 min	30 × (94°C 30 s / 60°C 30 s / 72°C 45 s)	72°C, 10 min	Shome et al. (2011)
Resistance genes	aadA	95°C, 15 min	35 × (94°C 1 min / 68°C 1 min / 72°C 1 min)	72°C, 10 min	Madsen et al. (2000)
	tetA, tetB	95°C, 15 min	35 × (94°C 1 min / 64°C 1 min / 72°C 1 min)	72°C, 10 min	Lanz et al. (2003)
	tetC	95°C, 15 min	35 × (94°C 1 min / 65°C 1 min / 72°C 1 min)	72°C, 10 min	
	sul1	95°C, 15 min	35 × (94°C 1 min / 68°C 1 min / 72°C 1 min)	72°C, 10 min	
	sul2	95°C, 15 min	35 × (94°C 1 min / 66°C 1 min / 72°C 1 min)	72°C, 10 min	
	sul3	95°C, 15 min	30 × (94°C 30 s / 51°C 30 s / 72°C 1 min)	72°C, 10 min	Perreten and Boerlin (2003)
	catI, floR, blaTEM, blaSHV	95°C, 15 min	30 × (94°C 30 s / 50°C 30 s / 72°C 90 s)	72°C, 10 min	Gow et al. (2008)
	ant(3ʺ)‐Ia (aadA1), dhfrI	95°C, 15 min	30 × (94°C 30 s / 50°C 30 s / 72°C 1 min)	72°C, 10 min	Ibekwe and Murinda (2011)
	dfrA1‐like	95°C, 15 min	30 × (94°C 30 s / 48°C 30 s / 72°C 1 min)	72°C, 10 min	
MLVA	10 VNTR loci (ccr001; cvn001–004, cvn007, cvn014–017)	95°C, 15 min	30 × (94°C 30 s / 60°C 90 s / 72°C 90 s)	72°C, 10 min	Løbersli et al. (2012)

To determine the size of the amplified fragments, the PCR products were analysed by agarose gel electrophoresis. Electrophoresis was performed on a 1.5% agarose gel stained with Safe‐Green Nucleic Acid Gel Stain (Applied Biological Materials Inc.) in 1× Tris‐borate‐EDTA (TBE) buffer. The gels were then examined under a UV transilluminator, and samples producing a band of the expected size for the target gene were considered positive.

### 2.6 Analysis of Antimicrobial Resistance Genes

PCR analysis was used to detect the antimicrobial resistance genes in the *E. coli* strains isolated from faecal samples of diarrheic calves, namely aadA, tetA, tetB, tetC, sul1, sul2, sul3, catI, floR, blaTEM, blaSHV, ant(3ʺ)‐Ia (aadA1), dhfrI and dfrA1‐like (Gow et al. 2008; Ibekwe and Murinda 2011; Lanz et al. 2003; Madsen et al. 2000; Perreten and Boerlin 2003). Primer sequences specific to the antimicrobial resistance genes and the expected PCR product sizes are provided in the Supporting Information. All antimicrobial resistance genes PCR reactions were performed using the HotStarTaq Master Mix Kit (Qiagen) according to the manufacturer's instructions. Each reaction was prepared to a final volume of 25 µL, comprising 12.5 µL of 2× HotStarTaq Master Mix, each primer at a final concentration of 0.5 µM, and approximately 100 ng of template DNA, with RNase‐free water added to bring the reaction to its final volume. The prepared reaction tubes were subjected to thermal cycling as specified in Table 1.

The antimicrobial resistance gene PCR products were analysed by electrophoresis on a 1.5% agarose gel stained with Safe‐Green Nucleic Acid Gel Stain (Applied Biological Materials Inc.) in 1× TBE buffer, and visualised under a UV transilluminator; samples showing a band of the expected size were considered positive.

### 2.7 Multiple‐Locus Variable‐Number Tandem Repeat Analysis

After completing the analyses of the isolated *E. coli* strains, including disc diffusion tests and the detection of virulence and antimicrobial resistance genes, 88 strains exhibiting multidrug resistance and a high number of virulence factors were selected for genotyping using the MLVA method. DNA extracts from the identified isolates were used to detect the target loci using the primers described by Lindstedt et al. (2007) and Løbersli et al. (2012) (Supporting Information). PCR was carried out for 10 specific loci (*ccr001, cvn016, cvn017, cvn001, cvn002, cvn003, cvn004, cvn007, cvn014 and cvn015)* in each strain to determine the Variable Number Tandem Repeat (VNTR) numbers, according to the reference methods. All MLVA PCR reactions were performed using the HotStarTaq Master Mix Kit (Qiagen) according to the manufacturer's instructions. Each reaction was prepared to a final volume of 25 µL, comprising 12.5 µL of 2× HotStarTaq Master Mix, each primer at a final concentration of 0.2 µM, and approximately 50 ng of template DNA, with RNase‐free water added to bring the reaction to its final volume. The prepared reaction tubes were subjected to thermal cycling as specified in Table 1. Locus sizes were determined based on the QIAxcel capillary gel electrophoresis system (Qiagen) using a Qiaxcel DNA High Resolution Kit. The number of repeat sequences at each tested locus was determined for each strain based on the PCR product sizes. The genotypic profiles of the strains were generated with PAST software using the repeat numbers at the 10 analysis loci, and the relationships among strains were determined using Ward's algorithm. The acquired data were then used to generate a dendrogram using iTOL software (Özgen et al. 2023; Letunic and Bork 2024).

### 2.8 OMV Analysis

Among the genotypic groups determined by the MLVA, two groups with large numbers of *E. coli* strains were selected, and 10 strains exhibiting high levels of antibiotic resistance and virulence were selected from each group (total 20) for OMV analysis.

Following the cultivation of the 20 selected *E. coli* strains in nutrient broth, OMVs were extracted using the ExoBacteria OMV Isolation kit according to the manufacturer's instructions. For the analysis of the OMVs based on their molecular weights, the extracted OMVs were mixed with a sodium dodecyl sulphate (SDS) loading buffer containing 5% 2‐mercaptoethanol, incubated at 100°C for 5 min on a dry heat block, and then analysed by polyacrylamide gel electrophoresis (PAGE). Following electrophoresis, the gels were stained with Coomassie Brilliant Blue (CBB) (Yonezawa et al. 2011).

### 2.9 Immunization of Experimental Animals

Strain selection was based on a representative sampling strategy to capture the diversity observed among isolates. For MLVA analysis, strains were selected to represent different virulence gene profiles and antimicrobial resistance patterns. A subset of strains was further chosen for OMV characterization to reflect the variability in protein profiles. The final strains were selected for further immunogenic evaluation based on a combination of genetic diversity, virulence traits and distinct OMV profiles, rather than using a predefined quantitative scoring system. For the immunization of experimental animals stage of the study, 5–7‐week old BALB/c mice were acquired and assigned to seven groups: Group I: Ec048 strain 1 × 10^9^, Group II: Ec048 strain 1 × 10^10^, Group III: Ec048 strain 1 × 10^11^, Group IV: Ec026 strain 1 × 10^9^, Group V: Ec026 strain 1 × 10^10^, Group VI: Ec026 strain 1 × 10^11^ and Group VII: negative control, with 10 animals immunized in each study group.

After completing the analysis specified within the scope of the project, the 20 tested strains were analysed and the two strains with the highest OMV content were identified. These two strains were then washed with phosphate‐buffered saline and centrifuged at 8000 × *g* for 15 min following cultivation in Luria–Bertani broth. The inactivation of *the E. coli* strains was carried out with a 0.4% formalin solution, after which the strains were incubated at 4°C for 18 h. The inactivated *E. coli* strains were then centrifuged at 4°C and 10,000 × *g* for 30 min. The resulting pellet was washed with PBS (Arshadi et al. 2020), and bacterial suspensions were then prepared at three different doses (1 × 10^9^, 1 × 10^10^, 1 × 10^11^) for the identification of the immune response elicited by the *E. coli* strains. The strains were inoculated onto sheep blood agar to verify inactivation, and the formalin‐inactivated bacterial suspensions were formulated with alum adjuvant (Arshadi et al. 2020).

For the immunization analysis, BALB/c mice in the experimental groups received two subcutaneous 0.1 mL injections of the inactivated *E. coli* strain, administered 21 days apart, to assess its immunogenic potential (Arshadi et al. 2020). The animals in the control group received two subcutaneous injections of 0.1 mL of physiological saline 21 days apart. Blood samples were collected on Day 0 (prior to the first inoculation), Day 21 (prior to the second inoculation) and Day 42 (21 days after the second inoculation) and analysed by flow cytometry.

### 2.10 Flow Cytometry Analysis

The parameters analysed by flow cytometry in the experimental group were CD4 (FITC), CD8 (APC), CD19 (PE/CY5), CD45 (PE/CY5), CD20 (APC), IL4 (APC), IL10 (FITC), IFN gamma (FITC) and TNF alpha (APC), as well as IgG (FITC) and IgM (PE/CY5) levels. A 100 µL blood sample was collected into an anticoagulant‐containing tube and placed on ice. The specific antibodies were diluted according to the manufacturer's protocols, and antibody consumption was optimized to ensure proper antibody performance. The antibody staining and flow cytometry analyses were prepared and validated. The antibody mixtures were transferred into 90 µL of blood and mixed, and the resulting solution was incubated on ice in the dark for 40 min. The solution was then vortexed following the addition of 2 mL RBC lysis solution, and then incubated in the dark for 10 min and centrifuged at 1100 rpm for 5 min. The supernatant was removed and the solution was vortexed following the addition of 1 mL standard wash solution. The solution was then vortexed again and incubated in the dark for 20 min following the addition of 500 µL stabilizing fixative. After centrifuging at 1100 rpm for 5 min, the supernatant was removed, and 1 mL of 2× wash solution was added and gently vortexed. Following centrifugation at 1100 rpm for 5 min, the supernatant was removed and 500 µL of standard wash solution was added to the suspension. The solution was then transferred to a flow cytometry tube and analysed using a flow cytometry device, and the results were depicted in graphs for subsequent comparison.

### 2.11 Statistical Analysis

Statistical analyses were performed using IBM SPSS Statistics (version 20, IBM Corp., Armonk, NY, USA). Data were expressed as mean ± standard deviation (SD). The normality of data distribution was assessed using the Shapiro–Wilk test.

Differences among experimental groups and time points were evaluated using two‐way analysis of variance (ANOVA), considering group and time as fixed factors. When significant effects were detected, multiple comparisons were performed using Tukey's post hoc test. A *p*‐value of < 0.05 was considered statistically significant. Graphs were generated using GraphPad Prism (version 11.0.0, GraphPad Software, San Diego, CA, USA).

## 3 Results

### 3.1 Collection of Study Samples and Bacteriological Culture

The faecal samples used in the study were collected from each province proportional to the regional cattle population. Accordingly, a total of 500 samples were collected from the studies provinces in the following numbers: Ağrı 75 (15.0%), Ardahan 75 (15.0%), Artvin 10 (2.0%), Bayburt 18 (3.6%), Erzincan 20 (4.0%), Erzurum 166 (33.2%), Gümüşhane 3 (0.6%), Iğdır 26 (5.2%) and Kars 107 (21.4%). In the bacteriological culture and isolation stage, *E. coli* was isolated and identified from 53.6% (268/500) of the faecal samples collected from diarrheic calves.

### 3.2 Antimicrobial Susceptibility Test and Analysis of Antimicrobial Resistance Genes

The disc diffusion method adopted for the antimicrobial susceptibility tests revealed the 268 *E. coli* isolates identified in the study to be resistant to enrofloxacin (52.5%), ampicillin/sulbactam (18.1%), trimethoprim/sulfamethoxazole (68.3%), amoxicillin/clavulanic acid (50.6%), oxytetracycline (88.3%), neomycin (35.5%), ceftiofur (10.2%) and marbofloxacin (46.4%). One *E. coli* strain isolated from a sample collected in the Kars province was found to exhibit resistance to all tested agents. The following antimicrobial resistance genes were identified in the *E. coli* isolates: *aada* (58.2%), *ant30* (21.2%), *tetA* (44.7%), *tetB* (6.5%), *tetC* (1.8%), *sul1* (8.2%), *sul*2 (21.2%), *sul3* (5.3%), *flor* (68.8%), *catl* (37.6%), *blatem* (25.9%), *blashv* (12.4%), *dhfa‐1like* (55.9%) and, *dhfrl* (56.5%). The phenotypic and genotypic resistance profiles of the *E. coli* isolates against amoxicillin/clavulanic acid, ampicillin/sulbactam, ceftiofur, trimethoprim/sulfamethoxazole, oxytetracycline and neomycin are presented in Table 2.

**TABLE 2:** Comparison of genotypic and phenotypic antimicrobial resistance in *Escherichia coli* strains isolated from calf diarrhoea cases.

Antibiotic	Phenotype	Genotype	% of dis‐agreement	% of agreement
Amoxicillin/clavulanic acid	S (*n:10*)	R (*n:10*)	32.1% (86/268)	67.9% (182/268)
	R (*n:76*)	S (*n:76*)		
Ampicillin/sulbactam	S (*n:37*)	R (*n:37*)	20.9% (56/268)	79.1% (212/268)
	R (*n:19*)	S (*n:19*)		
Ceftiofur	S (*n:53*)	R (*n:53*)	20.9% (56/268)	79.1% (212/268)
	R (*n:3*)	S (*n:3*)		
Trimethoprim/sulphamethoxazol	S (*n:46*)	R (*n:46*)	20.9% (56/268)	79.1% (212/268)
	R (*n:10*)	S (*n:10*)		
Oxytetracycline	S (*n:20*)	R (*n:20*)	32.8% (88/268)	67.2% (180/268)
	R (*n:68*)	S (*n:68*)		
Neomycin	S (*n:75*)	R (*n:75*)	31.0% (83/268)	69.0% (185/268)
	R (*n:8*)	S (*n:8*)		

Abbreviations: R: resistant; S: susceptible.

### 3.3 Determination of *E. coli* Virulence Genes

**FIGURE 1:**
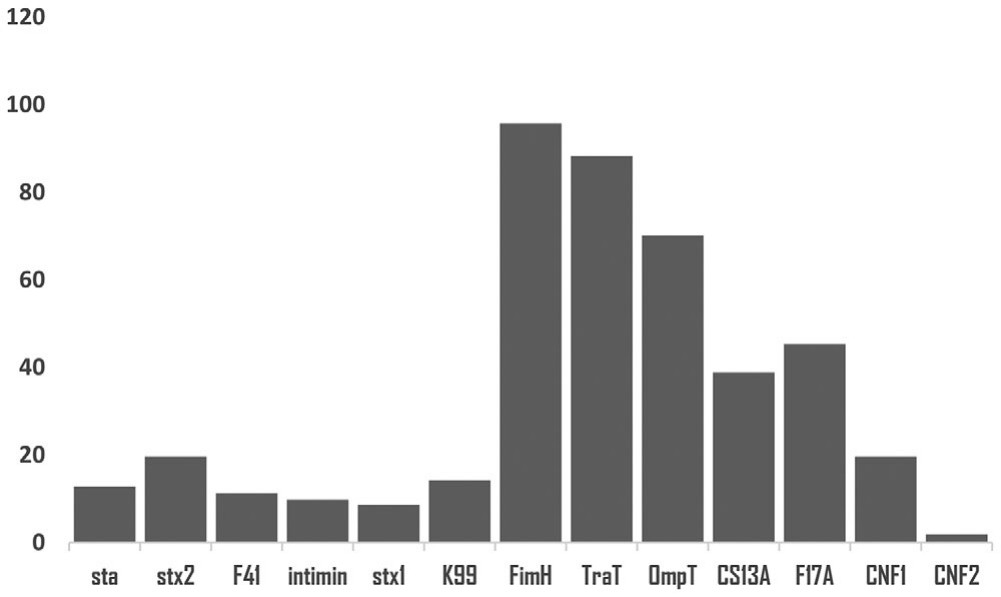
Percentage distributions of virulence genes detected in *Escherichia coli* isolates.

Virulence genes were analysed by PCR in conjunction with antimicrobial susceptibility testing. For this purpose, the following virulence factor gene regions were analysed: sta (12.7%), *stx2 (*19.4%), *f41* (11.2%)*, intimin* (9.7%), *stx1* (8.6%), *f5* (14.2%), *fimH* (94.8%), *traT* (87.3%), *ompT* (69.4%), *cs31A* (38.4%), *cnf1* (44.8%), *cnf2* (19.4%), *phoA* (51.9%) (Figure 1).

### 3.4 MLVA

**FIGURE 2:**
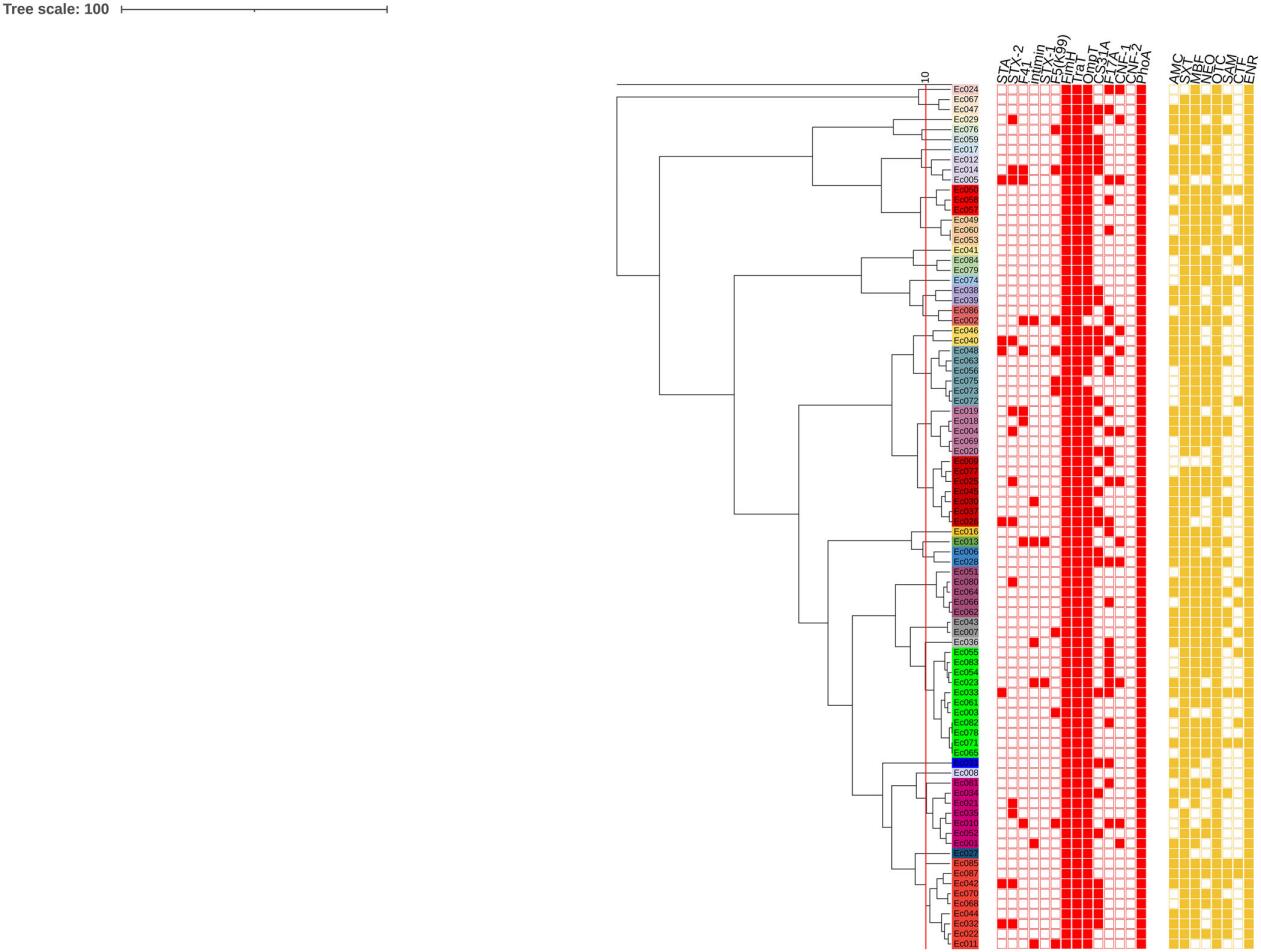
Dendrogram showing genotype groups, virulence genes and antimicrobial susceptibility of *Escherichia coli* isolates by MLVA.

A total of 88 *E. coli* isolates were selected for the MLVA based on the results of a virulence gene analysis and the antimicrobial susceptibility profiles. The MLVA identified 32 clonal groups exhibiting 10% similarity (Figure 2).

### 3.5 OMV Analysis

**FIGURE 3:**
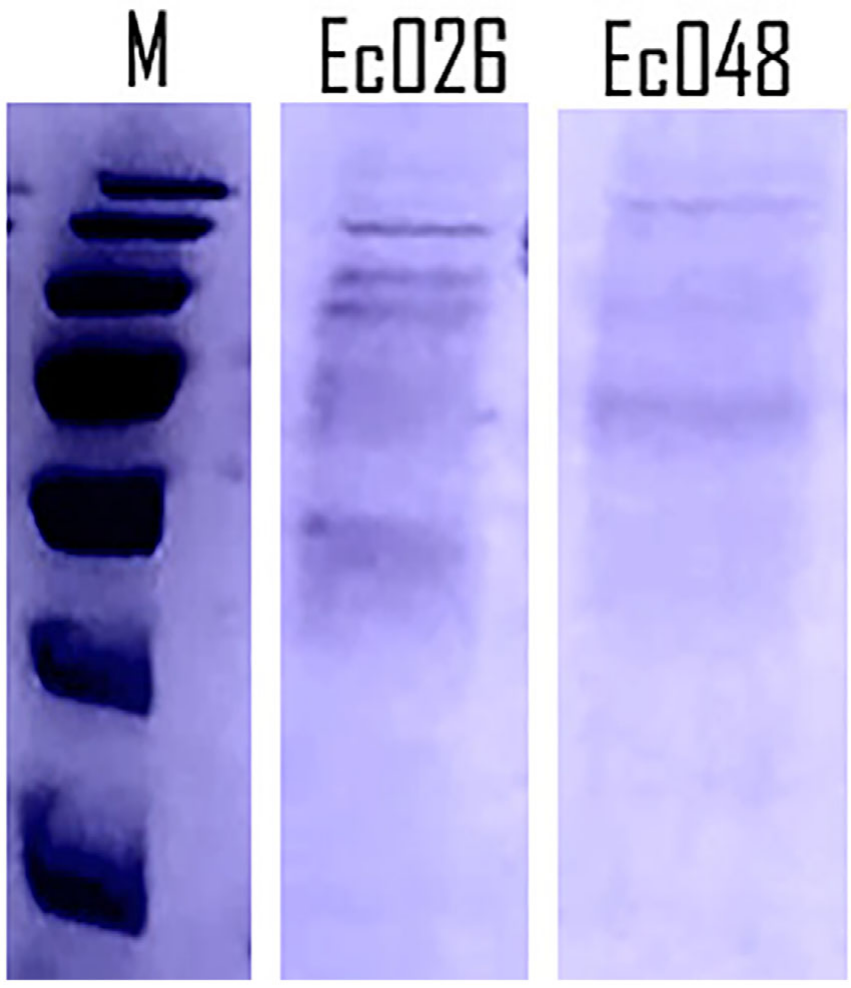
SDS‐Page profiles of OMVs in *Escherichia coli* strains.

The 20 *E. coli* isolates selected from the largest MLVA groups based on the findings of the MLVA analysis were then subjected to protein analyses, taking into account their virulence genes and antimicrobial resistance profiles. The isolates carrying the K99 fimbria were analysed for whole‐cell protein profiles, outer membrane proteins and OMVs (Figure 3). Based on the studies on protein profiles, the *E. coli* strains Ec048 and Ec026 were selected for inclusion in the experimental animal study, and the results of the virulence gene analysis and antimicrobial susceptibility tests for the two isolates are presented in Table 3.

**TABLE 3:** Virulence gene and microbial susceptibility analysis results of the preferred Ec026 and Ec048 strains as a result of virulence gene, antimicrobial susceptibility, MLVA, OMV analyses.

*E. coli* isolate	Amoxicillin/clavulanic acid	Trimethoprim/sulphamethox	Marbofloxacin	Neomycin	Oxytetracycline	Ampicillin/sulbactam	Ceftiofur	Enrofloxacin	Stx1	Stx2	F41	Intimin	Sta	K99	FimH	TraT	OmpT	CS31A	F17A	CNF1	CNF2	PhoA
***Ec026***	R	R	S	S	R	S	S	R	+	+	−	−	−	−	+	+	+	+	+	−	−	+
***Ec048***	R	R	R	R	R	S	S	R	+	−	+	−	−	+	+	+	+	+	−	+	−	+

*Note*: ‘+’ indicates virulence gene positive, ‘−‘ indicates virulence gene negative.

### 3.6 Inoculation of Experimental Animals and Flow Cytometry Analysis

**FIGURE 4:**
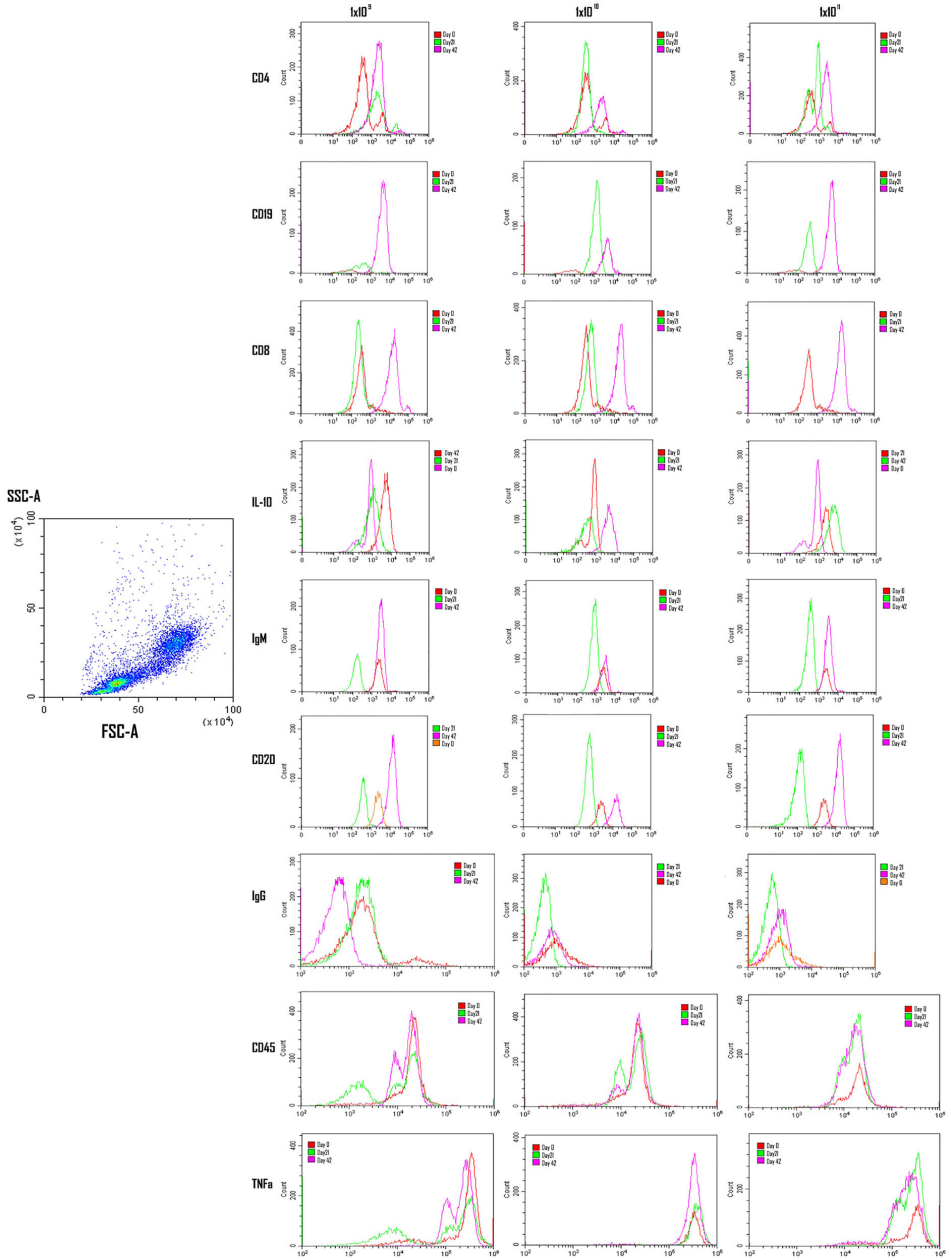
Flow cytometry measurements of changes in CD4, CD8, CD19, CD20, CD45, TNFalpha, IgM, IgG and IL‐10 values ​​in samples taken on Days 0, 21 and 42 in experimental animals of the Ec026 strain.

Blood samples collected from the experimental groups that received the selected *E. coli* strains and from the negative control groups on Days 0, 21 and 42 were analysed for CD4, CD8, CD19, CD20, IL4, IL10, IFN gamma, TNF alpha, IgG and IgM by flow cytometry (Figure 4).

**FIGURE 5:**
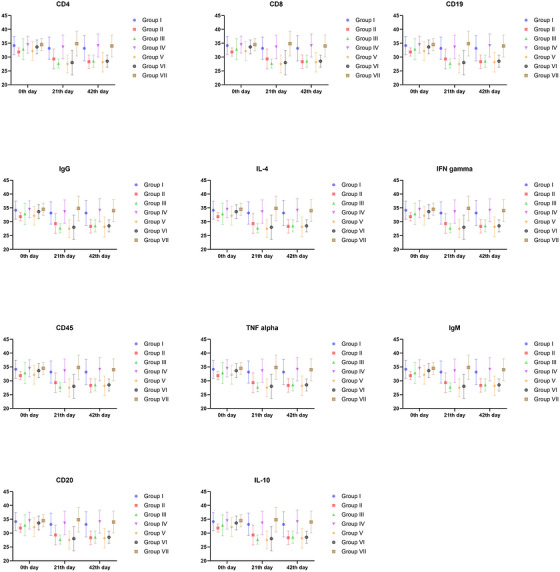
Changes in CD4, CD8, CD19, CD20, CD45, IgG, IgM, IL4, IL10, IFN gamma and TNF‐α levels across experimental groups (Group I–VII) at 0, 21 and 42 days post‐vaccination. Data are presented as mean ± SD.

CD45 levels were significantly affected by group, time and their interaction (*p* < 0.05). In particular, Groups III and VI exhibited a gradual increase from Day 0 to Days 21 and 42, which was significantly higher compared to the negative control group (Table 4). Similarly, TNF‐α and IgM levels showed significant effects of group, time and group × time interaction (*p* < 0.05), in Groups II, III, V and VI. CD20 expression was significantly affected by group and group × time interaction (*p* < 0.05) in Groups II, III, V and VI, while no significant effect of time alone was observed (*p* > 0.05). All groups (except the negative control group) showed a gradual increase in IFN gamma, CD19, IgG and IL4 levels, which were significantly influenced by group, time and their interaction (*p* < 0.05). For IL10, no significant effects of group or time were observed (*p* > 0.05); however, a significant group × time interaction was detected (*p* = 0.038), indicating differential regulation among groups over time. No statistically significant effects of group, time or group × time interaction were observed for CD4 and CD8 levels (*p* > 0.05) in any of the groups, and no gradual increase was noted from Day 0 to Days 21 and 42 (Figure 5).

**TABLE 4:** Analysis of variance results for immune parameters across different groups and time points. The effects of group, time (day) and group × time interaction were evaluated for CD4, CD8, CD19, CD20, CD45, IgM, IgG, TNF alpha, IL4, IL10, IFN gamma immune parameters.

Parameter	Effect	*F*	*p*	Partial *η* ^2^
CD4	Group	0.779	0.588	0.043
	Day	0.830	0.443	0.015
	Group × day	0.964	0.487	0.099
CD8	Group	1.406	0.219	0.074
	Day	1.121	0.329	0.021
	Group × day	0.836	0.612	0.087
CD45	Group	249.289	*p* < 0.05	0.934
	Day	188.146	*p* < 0.05	0.781
	Group × day	83.988	*p* < 0.05	0.905
TNF alpha	Group	138.472	*p* < 0.05	0.887
	Day	285.314	*p* < 0.05	0.844
	Group × day	46.637	*p* < 0.05	0.842
IgM	Group	158.190	*p* < 0.05	0.839
	Day	311.796	*p* < 0.05	0.855
	Group × day	45.626	*p* < 0.05	0.900
CD20	Group	2.760	*p* < 0.05	0.167
	Day	1.617	0.203	0.0298
	Group × day	3.525	*p* < 0.05	0.239
CD19	Group	37.768	*p* < 0.05	0.683
	Day	295.213	*p* < 0.05	0.849
	Group × day	15.412	*p* < 0.05	0.637
IgG	Group	12.392	*p* < 0.05	0.414
	Day	251.31	*p* < 0.05	0.827
	Group × day	15.702	*p* < 0.05	0.642
IL‐4	Group	54.116	*p* < 0.05	0.755
	Day	305.73	*p* < 0.05	0.853
	Group × day	18.141	*p* < 0.05	0.674
IL‐10	Group	0.518	0.792	0.028
	Day	0.025	0.974	4.842
	Group × day	1.934	0.038	0.181
IFN gamma	Group	95.782	*p* < 0.05	0.845
	Day	693.12	*p* < 0.05	0.929
	Group × day	26.605	*p* < 0.05	0.752

*Note*: *F*: *F* value; *p*: significance level; partial *η* ^2^: partial effect size.

## 4 Discussion

Calf diarrhoea remains a significant problem for the cattle breeding industry, affecting 5%–23% of farm calves stocks (Wilson et al. 2023). According to US data, 57% of calf deaths in the neonatal period are associated with calf diarrhoea (Cho and Yoon 2014). *E. coli* is the most commonly isolated pathogen in cases of calf diarrhoea (Mohammed et al. 2019). In the present study, the virulence, phenotypic and genotypic antimicrobial resistance profiles, MLVA‐based genotyping and OMV structures of *E. coli* strains associated with calf diarrhoea in the Eastern Anatolia Region of Türkiye, where cattle breeding is highly concentrated, are investigated. Based on the results of the analyses, two *E. coli* isolates showing immunogenic potential were selected, and the immune responses they elicited in experimental animals were evaluated.

Numerous studies in the literature have reported on the isolation of *E. coli* from cases of calf diarrhoea, identifying *E. coli* in 43.5–100.0% of the investigated cases (Mohammed et al. 2019; Coşkun and Şahin 2023; Cengiz and Adıgüzel 2020; Haydardedeoğlu et al. 2023; Kirkan et al. 2018; Nguyen et al. 2011). In the present study, *E. coli* was isolated in 53.6% (268/500) of the faecal samples collected from diarrheic calves, consistent with the findings of similar studies. The rate of *E. coli* isolation from diarrheic calves varies from year to year and across different geographic regions. This finding can be attributed to the lack of thorough assessment of the aetiology in cases of diarrhoea and to whether the isolation was performed during the treatment phase.

Diarrhoeagenic *E. coli* strains must possess virulence factors to cause diarrhoea in calves (Ryu et al. 2020). The main virulence factors reported in *E. coli* strains isolated from calf diarrhoea cases include fimbrial adhesion factors (*f41, f17, fimA, fimC, f5*), outer membrane adhesion factor (intimin), Shiga toxins and enterotoxins (STa) (Cengiz and Adıgüzel 2020; Nguyen et al. 2011; Jia et al. 2022; Salvadori et al. 2003). STEC strains are commonly isolated in cases of calf diarrhoea, but are reported primarily as a public health concern rather than a major challenge in animal husbandry (Ryu et al. 2020). Consistent with earlier studies in the literature, the present study revealed toxin, haemolysin and fimbrial virulence factors in *E. coli* strains to be associated with the aetiology of calf diarrhoea.

The primary treatment protocols for calf diarrhoea often include antibiotics and antibiotic combinations; however, the continuous and indiscriminate use of antibiotics has led to a progressively increasing antimicrobial resistance spectrum in *E. coli* strains (Jia et al. 2022). Studies have reported variable resistance rates to antibiotics among the *E. coli* strains associated with calf diarrhoea, including trimethoprim/sulfamethoxazole (25.0%–91.7%), neomycin (46.2%–60.9%), oxytetracycline (33.3%–80.0%), enrofloxacin (5.6%–41.3%), marbofloxacin (49.6%), ampicillin/sulbactam (11.3%–86.7%), ceftiofur (7.5%) and amoxicillin/clavulanic acid (12.0%–33.0%) (Mohammed et al. 2019; Güler et al. 2008; Coşkun and Şahin 2023;Cengiz and Adıgüzel 2020; Haydardedeoğlu et al. 2023; Kirkan et al. 2018). The antimicrobial agent resistance profiles identified in the present study are similar to those reported in earlier studies. The analysis of antimicrobial resistance genes in the present study revealed that the phenotypic and genotypic resistance profiles were not fully concordant (Table 2). The phenotypic susceptibility of strains with antimicrobial resistance genes can be attributed to the need for an integron promoter in their expression. That said, the phenotypic resistance of strains that lack resistance genes suggests the potential for the emergence of new resistance factors (Abdelwahab et al. 2022).

MLVA can reveal the differences between the bacterial strains responsible for outbreaks, and offers superior discriminatory power over PFGE approaches. Kumar et al. (2014) conducted an MLVA to investigate the genotypic variations of non‐O157 STEC strains isolated from faecal samples of humans and animals, as well as from meat samples in India, and revealed 19 genotypic variations of non‐O157 STEC strains. Noller et al. (2003), on the other hand, used MLVA to identify 64 genotypes among 80 *E. coli* O157‐H7 strains. In a study of 10 cattle farms in Sweden, Duse et al. (2016) identified 24 MLVA genotypes among the 136 collected quinolone‐resistant *E. coli* isolates. Furthermore, Krüger et al. (2015) conducted an MLVA and revealed 19 different genotypes among the 29 *E. coli* O26:H11 serotypes isolated in different years. MLVA genotyping is particularly appropriate for outbreak‐typing, and can be beneficial in identifying different clonal lineages within strains of various serogroups as a cost‐effective, rapid and inter‐laboratory compatible approach.

Adjuvants containing aluminium salts are commonly employed in the formulation of vaccines, and generally elicit Th2 immune response, and more rarely, cellular immune response (Hu et al. 2010). Schaut et al. (2019) reported an increase in IL‐4, IgG and IFN gamma levels and a decrease in IL‐10 levels, determined by flow cytometry, following the administration of an inactive *E. coli* strain to mice with adjuvant compared to a vaccine formulation without adjuvant. In the study by Gohar et al. (2016), BALB/c mice were subcutaneously immunized with a combined *E. coli* vaccine candidate, resulting in 100% survival following challenge, indicating strong protective efficacy. The immunization elicited a significant increase in specific systemic IgG, IFN‐γ and IL‐6 levels, particularly during the first 2 weeks post‐immunization. In the study by Zhao et al. (2022), mice immunized with *E. coli* antigens showed significantly higher specific IgG levels in serum compared to controls. Immunization also led to TNF‐α concentrations. These findings are consistent with our results, which demonstrated that immunization with selected *E. coli* strains elicited robust humoural immune responses, particularly IgG, and suggest that combined or multivalent formulations may enhance early immune activation, supporting the rationale for further evaluation in target species.

The present study revealed an increase in the markers responsible for humoural immune response (i.e., IgG, IgM, CD20) in the experimental animals; however, no significant change was observed in such cellular immune response markers as CD4 and CD8. The increase in IL‐4 levels and the accompanying reduction in IL‐10 levels suggest the triggering of a humoural immune response (Awate et al. 2013; Guthmiller et al. 2017). Across the experimental groups, the administration of *E. coli* Ec026 and Ec048 isolates at doses of 1 × 10^10^ and 1 × 10^11^ elicited a more robust immune response than the 1 × 10^9^ dose and the negative control group. CD45 is used to determine the activation of T and B lymphocytes (Božić et al. 2002). In the present study, the greatest increase in CD45 levels was observed in groups that received a 1 × 10^11^ dose of the Ec026 and Ec048 isolates. Within the present study, the 1 × 10^11^ dose of the *E. coli* strains elicited the optimal immune response, while the immune response of the 1 × 10^10^ dose could be considered adequate. The 1 × 10^9^ dose, on the other hand, failed to trigger an adequate immune response.

The mouse model is a simple and economical alternative for testing vaccines against various bovine diseases because it overcomes disadvantages such as the high costs and facility requirements associated with using large animals (Gnazzo et al. 2020). To maintain optimal health in a cattle herd, it is essential to understand natural infection‐preventing immune mechanisms and how management strategies such as vaccination, biosecurity, nutrition, breeding and calf care can enhance immune protection, particularly considering the critical role of colostrum in providing neonatal immunity in calves (Vlasova and Saif 2021). Colostrum‐derived immunoglobulins, especially IgG, play a central role in passive immunity in neonatal calves (Pari et al. 2025). Although our findings in the murine model demonstrate promising humoural immune responses, further studies in the target species are required to confirm the translational relevance of these results.

One limitation of this study is the absence of a positive control group. Since the primary objective was to comparatively evaluate the immunogenic characteristics of different candidate preparations rather than to assess protective efficacy against a commercial vaccine, no suitable reference vaccine or strain representing regional field conditions was available. Therefore, immune responses were interpreted relative to the negative control group and among experimental groups. Future studies including a well‐defined positive control would be valuable to further validate the protective potential of the selected candidate. A second limitation of this study is the absence of a positive control group for the flow cytometry analysis, which restricted the interpretation of immune responses to comparisons with the negative control and among experimental groups rather than against a standardized reference.

## 5 Conclusion

In conclusion, the Ec026 and Ec048 *E. coli* strains, commonly isolated from the study area, exhibited virulence characteristics and antimicrobial susceptibility profiles that support their consideration as strains with immunogenic potential for further evaluation in studies aimed at controlling calf diarrhoea. Field trials of the two identified strains in the target animal population, and a comparison with commercially available vaccines will support the generalization of the findings of the present study. Future studies in neonatal calves, including the evaluation of maternal immunity and challenge experiments, are required to confirm the protective potential of the selected strains.

## Author Contributions

**Yasemin Erdoğan**: conceptualization, investigation, funding acquisition, writing – original draft, methodology, formal analysis, data curation, validation, visualization, writing – review editing. **Ediz Kağan Özgen**: conceptualization, investigation, funding acquisition, writing – original draft, methodology, validation, visualization, writing – review and editing, formal analysis, project administration, data curation, supervision, resources. **Murat Özmen**: methodology, validation, formal analysis, investigation, writing – original draft. **Elif Karadeniz Pütür**: investigation, validation, formal analysis, writing – original draft. **Nebahat Polat**: methodology, investigation, validation, formal analysis, writing – original draft. **Ersan Pütür**: methodology, investigation, validation, formal analysis, writing – original draft. **Mücahit Harmancı**: investigation, validation, methodology, formal analysis, writing – original draft. **Ercan Atalay**: investigation, validation, formal analysis, methodology, writing – original draft. **Ahmet Temur**: investigation, formal analysis. **Meryem Güloğlu**: investigation, validation, formal analysis, methodology, writing – original draft. **Lütfullah Celep**: investigation, methodology, validation, formal analysis, writing – original draft. **Seyda Cengiz**: methodology, investigation, writing – original draft. **Mehmet Cemal Adıgüzel**: methodology, investigation, writing – original draft. **Cihan Öz**: methodology, investigation, writing – original draft, validation, visualization, formal analysis. **Timur Gülhan**: methodology, investigation, writing – original draft. **Alper Çiftci**: methodology, investigation, writing – original draft. **Erhan Çiftgül**: methodology, writing – original draft.

## Funding

This study was supported by the Republic of Türkiye Ministry of Agriculture and Forestry General Directorate of Agricultural Research and Policies (Project Number: TAGEM/HSGYAD/B/22/A5/P1/5112).

## Ethics Statement

This study was conducted with the approval of the Erzurum Veterinary Control Institute Animal Experiments Local Ethics Committee (decision no: 13067196‐53, dated 9 November 2020).

## Conflicts of Interest

The authors declare no conflicts of interest.

## Supporting information




**Supporting File 1**: vms371151‐sup‐0001‐SupMat‐File.xlsx

## Data Availability

The datasets generated during and/or analysed during the current study are available from the corresponding author on reasonable request.
